# Professional identity influence mechanism of students majoring in mining with a view to sustainable development

**DOI:** 10.3389/fpsyg.2025.1581840

**Published:** 2025-06-19

**Authors:** Shijie Gao, Xigui Zheng, Ying Wang

**Affiliations:** ^1^School of Public Administration, China University of Mining and Technology, Xuzhou, China; ^2^School of Mines, China University of Mining and Technology, Xuzhou, China

**Keywords:** professional identity, sense of fulfillment, personal behavior, structural equation model, education

## Abstract

**Introduction:**

This study explores the mediating role of behavioral engagement in the relationship between professional identity (PI) and sustainable competence among mining engineering students.

**Methods:**

Drawing on a structural equation model (SEM), the research investigates how PI influences students’ sense of fulfillment (SF) through personal behavior (PB). Data were collected from 1,007 mining students across China.

**Results:**

The findings reveal that PI significantly enhances SF both directly (β = 0.562, *p* < 0.001) and indirectly through PB (β = 0.425, *p* < 0.001). While environmental, peer, and curriculum factors were hypothesized to influence PI, their direct effects were not statistically significant.

**Discussion:**

The study underscores the critical role of PI in fostering sustainable competence and highlights PB as a key mediator in this process. These results offer valuable insights for educational institutions aiming to cultivate professional identity and sustainable development among students in high-risk industries like mining. Practical recommendations include strengthening PI education, fostering positive PB, and optimizing external cultural and curricular environments to enhance students’ academic and professional outcomes.

## 1 Introduction

Sustainable development has emerged as a defining theme of our era. Regardless of whether it is interpreted as an immutable truth (Redcliffe, 1987) a paradox (O'Riordan, 1985), or an evolutionary process (Holmberg, 1992), its core fundamentally resides in practice (Kates et al., 2001), with the ultimate aim of ensuring the sustainable development of humanity. In the knowledge economy era, societal progress is primarily driven by ongoing knowledge innovation and rapid technological industrialization, both of which rely on a steady and evolving talent pool (Ibarra, 1999). As college students constitute the primary drivers of sustainable development, it is imperative that their education undergoes rigorous exploration and proactive implementation.

The rapid evolution of Industry 4.0 and Education 4.0 has significantly reinforced the importance of sustainable development as a defining theme. As Chakraborty et al. (2023) demonstrate in their mapping of Industry 4.0 and Education 4.0, the integration of advanced technologies and educational paradigms creates new pathways for sustainable practices. Furthermore, Chakraborty et al. (2024) highlight how sustainability perception among students, particularly in resource-intensive industries like petroleum processing, becomes crucial for Society 5.0’s emergence. These studies underscore that sustainable development in the mining industry is not merely an environmental concern but a comprehensive framework that integrates technological advancement, educational innovation, and social responsibility.

**Figure 1:**
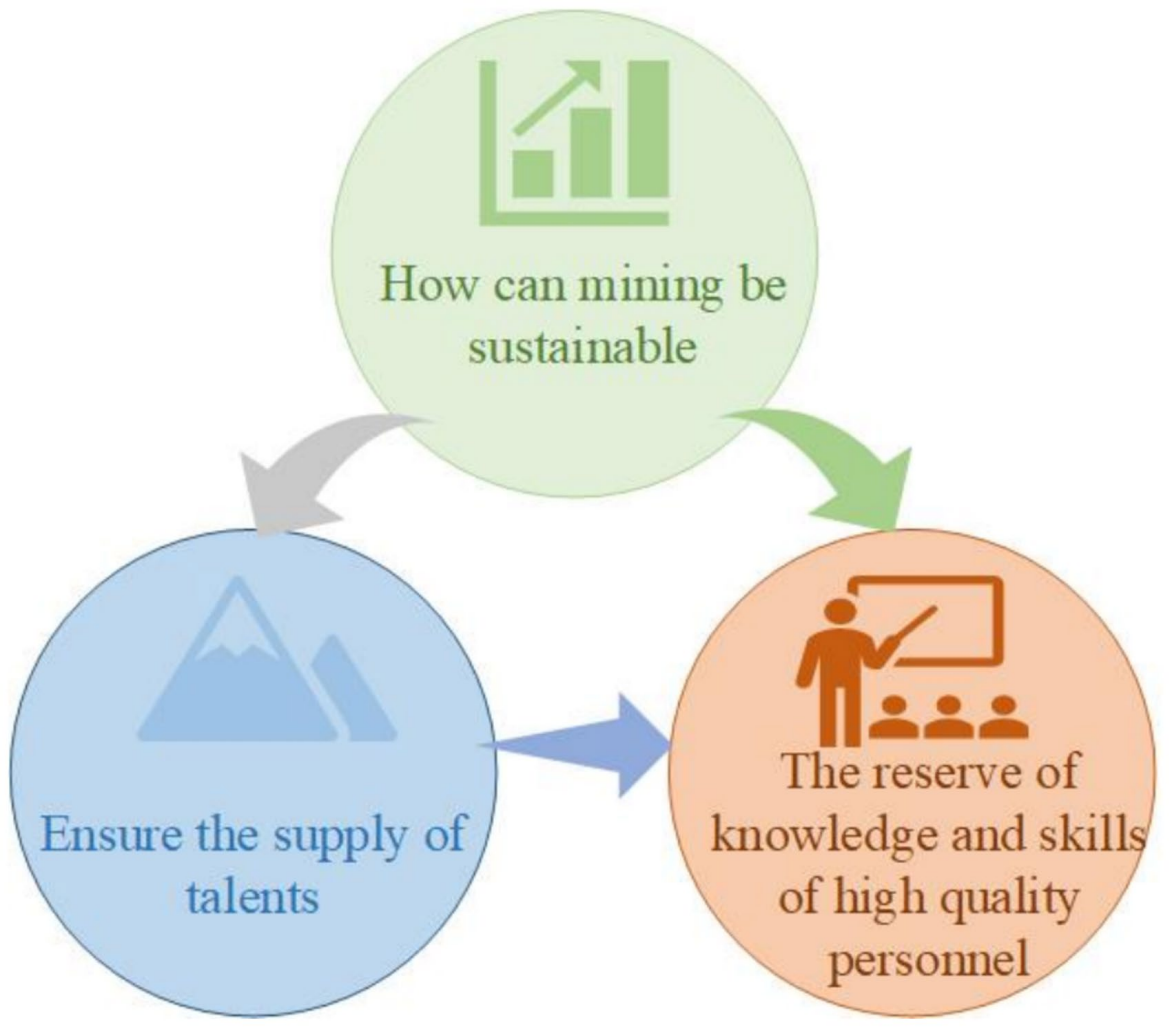
The relationship of mining sustainable development.

Mining, a traditionally challenging and risky industry, is frequently dismissed as a “sunset industry.” Yet, with the forces of intensified global competition coupled with mounting resource and environmental constraints, the mining sector is confronted with a critical dilemma. On one hand, there is an increasing demand for talents who are not only specialized but also capable of continuous knowledge development. On the other hand, the harsh working conditions and high safety risks inherent in the mining industry have contributed to an acute talent shortage, thereby constraining its sustainable development. In this context, professional identity (PI) emerges as a pivotal factor that influences students’ learning motivation, sense of fulfillment (SF), and ultimately the industry’s sustainable growth. The framework depicting the sustainable development of the mining sector is presented in Figure 1.

The rapid evolution of Industry 4.0 and Education 4.0 has significantly reinforced the importance of sustainable development as a defining theme. As Chakraborty et al. (2023) demonstrate in their mapping of Industry 4.0 and Education 4.0, the integration of advanced technologies and educational paradigms creates new pathways for sustainable practices. Furthermore, Chakraborty et al. (2024) highlight how sustainability perception among students, particularly in resource-intensive industries like petroleum processing, becomes crucial for Society 5.0’s emergence. These studies underscore that sustainable development in the mining industry is not merely an environmental concern but a comprehensive framework that integrates technological advancement, educational innovation, and social responsibility.

In the mining industry context, this emergent sustainability theme becomes particularly crucial given the sector’s environmental impact and resource management challenges.

PI is defined as a “relatively stable and enduring configuration” of individuals in their professional roles, or alternatively, as “a dynamic process of continuous construction and maintenance” (Beijaard et al., 2000). It encapsulates a unique psychological attachment (Henning, 2001), serves as an ideological tool (Margo, 2002), and reflects an emotional orientation (Roderick, 2003), which is evidenced by the degree of affective connection that students experience toward their academic majors. This connection is inherently dynamic, varying with individual cognitive and emotional developments, and in turn, manifests in behavioral differences influenced by both environmental factors and internal processes.

Meanwhile, the term “sense of fulfillment” in the Chinese context primarily assesses personal satisfaction and happiness (Diener, 1984), sharing conceptual similarities with constructs such as “happiness,” “subjective well-being,” and “life satisfaction.” Essentially, SF is a “psychological feeling” that is elicited by one’s objective achievements or subjective gains (Beijaard et al., 2000) and as it originates from positive “gain” rather than “loss,” it naturally bears a positive valence (Huang and Wu, 2017). In academic discourse, SF can be equated with the experience of gaining or seen as an integration of both the content and the experiential aspects involved in the process of gaining (Zheng and Chen, 2017; Dong et al., 2019). Collectively, PI and SF form a conjugate mapping relationship; students who exhibit a stronger professional identity with respect to their majors tend to experience higher levels of fulfillment, a phenomenon that ultimately contributes to the sustainable development of the mining industry.

Within this context, this study focuses on students majoring in mining. Given the close interrelation and significant explanatory power of PI and SF, these two variables are employed to construct an influence mechanism model. Specifically, as individuals cultivate a professional identity toward their majors, this identity stimulates personal initiative and motivates them to engage in proactive behaviors, which in turn fosters a sense of fulfillment. Based on this rationale, personal behavior (PB) is introduced as a mediating variable, and a structural equation model (SEM) is utilized to examine the differential effects of PI on PB, of PB on SF, and of PI on SF. The findings of this research are expected to enrich the theoretical framework of sustainable development in education and provide robust empirical evidence for the sustainable development of mining talents.

## 2 Literature review

### 2.1 Measurement and influencing factors of PI

#### 2.1.1 Measurement of PI

PI is a multidimensional construct that has been conceptualized and operationalized in various ways. The existing literature categorizes PI into cognitive, evaluative, and behavioral dimensions (Shelley, 2004). Despite the lack of a universally consistent measurement scale, researchers have developed frameworks tailored to specific research objectives. These frameworks can be broadly classified into one-dimensional, two-dimensional, multi-dimensional, and extended theories.

The one-dimensional theory posits that PI is essentially the positive emotions individuals exhibit when they accept and identify with a professional entity to perform work (Roderick, 2003). The two-dimensional theory expands this view by incorporating implicit attitudes and explicit behaviors as the two core dimensions of PI (Aranya and Frris, 1984). The three-dimensional theory further refines this by measuring PI through professional cognition, emotion, and attitude (Wang and Liu, 2007), while the four-dimensional theory provides a more comprehensive framework, encompassing cognition, emotion, behavior, and relevance (Zhang et al., 2008). Some scholars have also proposed a five-dimensional theory (Li et al., 2011). Among academic research, the dimensionality of professional identity measurement (whether unidimensional or multidimensional) should be guided by the research objectives and theoretical framework, with the primary criterion being the ability to comprehensively capture the construct from multiple angles and reflect its essential characteristics.

To account for the inherent complexity of PI, some studies have introduced additional dimensions, such as professional value identity and professional expectation, to better capture the dynamic and emotional nature of PI. These extensions have enhanced the theoretical and practical utility of PI frameworks.

#### 2.1.2 Influencing factors of PI

The antecedents of PI can be broadly categorized into internal and external factors. Internal factors include individual needs, personal characteristics, values, and psychological traits, while external factors encompass workplace environment, social status, and organizational characteristics.

Henning (2001) identified personal characteristics as a critical influencing factor, finding that individuals with higher extroversion and openness to new experiences tend to exhibit stronger PI. Nehami (2004) highlighted the importance of the relationship between individuals and their professions, professional competence, the alignment between personal needs and professional responsibilities, and workplace challenges. Yazdannik et al. (2012) emphasized the role of social status, professional prestige, respectability, and salary level in shaping PI.

Recent studies have also explored the role of personality traits and academic factors. For instance, Hu and Huang (2006) conducted an MBTI personality test on college students and found that specific indicators within personality dimensions can predict PI. Cross (2006) identified gender and academic achievement as significant predictors of PI among students at various educational stages. Li et al. (2011) further demonstrated that students who independently choose their majors tend to have higher internal motivation, stronger self-efficacy, and thus higher PI.

Moreover, external interventions such as strengthening social practice (Hamilton, 2013), transforming educational models (Slivia and Edoardo, 2009), and improving curriculum systems (Shelley, 2004) have been identified as effective strategies for enhancing PI. These findings collectively underscore the complexity of PI’s antecedents and the need for a holistic approach to its cultivation.

### 2.2 Measurement and influencing factors of SF

#### 2.2.1 Measurement of SF

The measurement of SF has evolved significantly, with researchers employing diverse frameworks to capture its multifaceted nature. Kozma and Stones (1980) developed the Memorial University of Newfoundland Scale of Happiness, which assesses subjective happiness across short-term and long-term emotional dimensions. Diener (1984) proposed a framework that evaluates SF through life satisfaction and subjective emotional experiences, encompassing both positive and negative emotions.

In the context of education, SF has been conceptualized similarly to academic self-efficacy and sense of academic achievement, with dimensions such as sense of accomplishment and positive emotion. Wood and Locke (1987) developed the Academic Self-Efficacy Questionnaire to measure students’ self-efficacy in confronting various academic tasks. Pintrich and De (1990) designed a questionnaire composed of two dimensions: self-efficacy in learning behavior and self-efficacy in learning ability. Jinks and Morgan (1999) introduced the Student Efficacy Scale, which measures SF through three dimensions: aptitude, situation, and effort. Pajares and Valiante (1997) constructed a scale focused on students’ self-efficacy in writing, while the University of California (Douglass et al., 2014) measured SF based on students’ self-evaluation.

SF has also been assessed through satisfaction-based frameworks. Zhou et al. (2016) designed a Likert 5-point scale based on the National Survey of Student Satisfaction, incorporating five dimensions: identification, satisfaction status, participation opportunity, and achievement level. Chen et al. (2022) developed a Likert 7-point scale for college students’ SF, comprising five dimensions: positive experience, academic achievement, interpersonal sentiment, practical participation, and personal growth. Li et al. (2022) compiled a Likert 5-point scale for postgraduate students’ SF, which includes five dimensions: supervisor’s affinity, hope, friendliness, self-confidence, and pleasure. Academic burnout has also been used as a contrasting index for SF measurement (Pan, 2014).

#### 2.2.2 Influencing factors of SF

The antecedents of SF can be broadly categorized into internal and external factors. Internal factors include learning motivation, learning stress, academic self-efficacy, learning engagement, and learning strategy. Catherine (Frédéric et al., 2010) found that autonomous learning motivation is significantly correlated with academic achievement and acts as a mediator between academic self-efficacy and academic achievement. Anna et al. (2005) demonstrated the applicability of academic self-efficacy in predicting academic achievement. Frank et al. (2010) highlighted the impact of parenting styles on academic self-efficacy.

External factors, such as employment security and career services, have also been identified as significant influencers. Ouyang et al. (2018) confirmed that employment security and career services noticeably influence college students’ SF. Zhang and Hao (2017) found that emotional bonds and interpersonal trust at the social level exert a significant positive effect on college students’ SF. Additionally, psychological qualities and cognitive biases play a role in shaping SF. For instance, students with high SF are better at regulating negative emotions (Rogaten and Moneta, 2017), and ideal academic performance fosters positive emotions (Putwain et al., 2013). Conversely, students with low SF are more prone to academic frustration, which may lead to internet addiction and other psychological issues (Wu et al., 2023). Furthermore, extroverted personality traits have been linked to a higher propensity for experiencing happiness (Headey and Wearing, 1989).

## 3 Model construction

SEM, a multivariate statistical technique integrating factor analysis and path analysis, is widely applied in fields such as social sciences, psychology, and management to explore complex relationships between variables. This model involves multiple variable types: endogenous variables, analogous to dependent variables in regression analysis, are influenced by other variables; exogenous variables, equivalent to independent variables, can affect other variables but are not reciprocally influenced; mediating variables act as bridges between independent and dependent variables to explain the mechanism of variable interactions; and moderating variables influence the direction or strength of the relationship between independent and dependent variables. By constructing path diagrams to visually represent variable relationships and combining methods such as confirmatory factor analysis and path analysis, SEM can test direct and indirect relationships between variables, analyze mediating and moderating effects, and effectively handle multiple dependent variables, measurement errors, and latent variables. This makes it a powerful tool for in-depth exploration of variable relationships.

### 3.1 Methodological innovation and assumptions

To our knowledge, this study represents one of the first attempts to integrate personality and interpersonal traits within a mathematical construct using SEM in the context of mining education. While previous studies have examined professional identity and fulfillment separately (Wang and Liu, 2007; Chen et al., 2022), the integration of these psychological constructs with behavioral mediators in a unified mathematical framework is novel.

Key assumptions underlying this approach include:

1 Professional identity and sense of fulfillment can be quantified through self-reported measures.
2 The relationships between variables are linear and can be captured through structural equations.
3 Cultural and contextual factors specific to Chinese mining education are generalizable.

Potential limitations include:

1 Self-report bias in measuring psychological constructs.
2 The cross-sectional nature of data limiting causal inference.
3 Potential unmeasured confounding variables affecting the relationships.

### 3.2 Variable determination

Human sustainable development is influenced by a combination of natural, social, and individual factors. In the context of mining education, the factors influencing PI from a sustainable development perspective include environmental factors, peer factors, curriculum factors, and PB. This study investigates the relationship between PI and SF by designating PI, environmental factors, peer factors, and curriculum factors as independent variables, SF as the dependent variable, and PB as the mediating variable.

#### 3.2.1 Determination of independent variables

PI is defined as the acceptance of professional knowledge and skills, identification with professional values and norms, and the interest or tendency to actively engage in learning and exploring one’s major with a positive attitude and proactive behavior.

Environmental factors encompass the social, campus, and family environments that directly or indirectly influence an individual’s learning experiences, psychological states, and behavioral manifestations. Peer factors focus on interactions and relationships with peers in academic and daily life, including teacher-student and peer-peer dynamics, which significantly impact learning motivation, attitudes, and PI. Curriculum factors refer to the teaching activities and content designed by institutions to achieve educational objectives, including curriculum design, course content, and assessment methods, all of which directly affect students’ learning experiences and outcomes.

Through a review and integration of existing literature, this study identifies the influencing factors and measurement indicators of PI. Regarding measurement indicators, given the diverse dimensional divisions of PI in current research and the focus of some studies on the internal appropriateness and emotionality of identity connotations, this study selects professional interest, professional belongingness, and professional values as measurement indicators for the degree of PI, comprehensively considering the cognitive, emotional, and value dimensions of the profession. In terms of influencing factors, drawing on existing research that explores influencing factors from implicit and explicit perspectives and combining analyses of key factors such as environmental and individual factors, environmental factors, peer factors, and curriculum factors are incorporated into the category of influencing factors. Among them, environmental factors correspond to the influence of the external environment, peer factors are associated with interactions between individuals, and curriculum factors align with strategies to improve the curriculum system to enhance PI.

#### 3.2.2 Determination of the dependent variable

SF is a dual-natured construct, reflecting both subjective and objective aspects. It captures the psychological state of surveyed students and the psychological experiences derived from knowledge acquisition, skill development, emotional value manifestation, and personal growth throughout their academic journey. SF is categorized into four dimensions: sense of achievement, positive emotion, self-improvement, and resource availability.

Through a systematic review and analysis of existing literature, the measurement indicators of SF were identified. Based on the connotative relationships among academic gain, academic self-efficacy, and academic achievement, this study drew on the dimensional design concepts of multiple mature scales. For example, it referenced the measurement of self-efficacy in different academic contexts from academic self-efficacy questionnaires, as well as the division of dimensions such as achievement and emotion in various satisfaction and gain scales. Ultimately, four measurement indicators were refined: achievement realization, positive emotional experience, self-enhancement perception, and resource possession awareness.

#### 3.2.3 Determination of the mediating variable

Mediating variable refers to a variable that lies between the independent variable and the dependent variable in a causal relationship, serving as a bridge or intermediate link. It is not only influenced by the independent variable but can also affect the dependent variable, meaning that the independent variable acts on the dependent variable by influencing the mediating variable. Theoretically, this explains the internal mechanisms and pathways through which the independent variable affects the dependent variable.

Regarding the study on the impact of professional identity on SF, a comprehensive review of the existing research in Section 2.2 reveals that students’ PI cannot directly promote SF. Instead, it typically acts indirectly on academic gain by influencing internal factors such as individual learning motivation and self-efficacy. In this study, such factors are collectively termed “Personal Behavior.” This factor plays the role of a mediating variable in the impact of PI on SF. PB refers to an individual’s responses and manifestations within specific environments and situations, grounded in their inherent cognition, emotion, motivation, and external stimuli. For mining students, PB involves specific behaviors in professional study, practical activities, interpersonal interactions, and other contexts, reflected in learning attitudes, study strategies, teamwork collaboration, and problem-solving capabilities.

### 3.3 Research hypotheses

**Figure 2:**
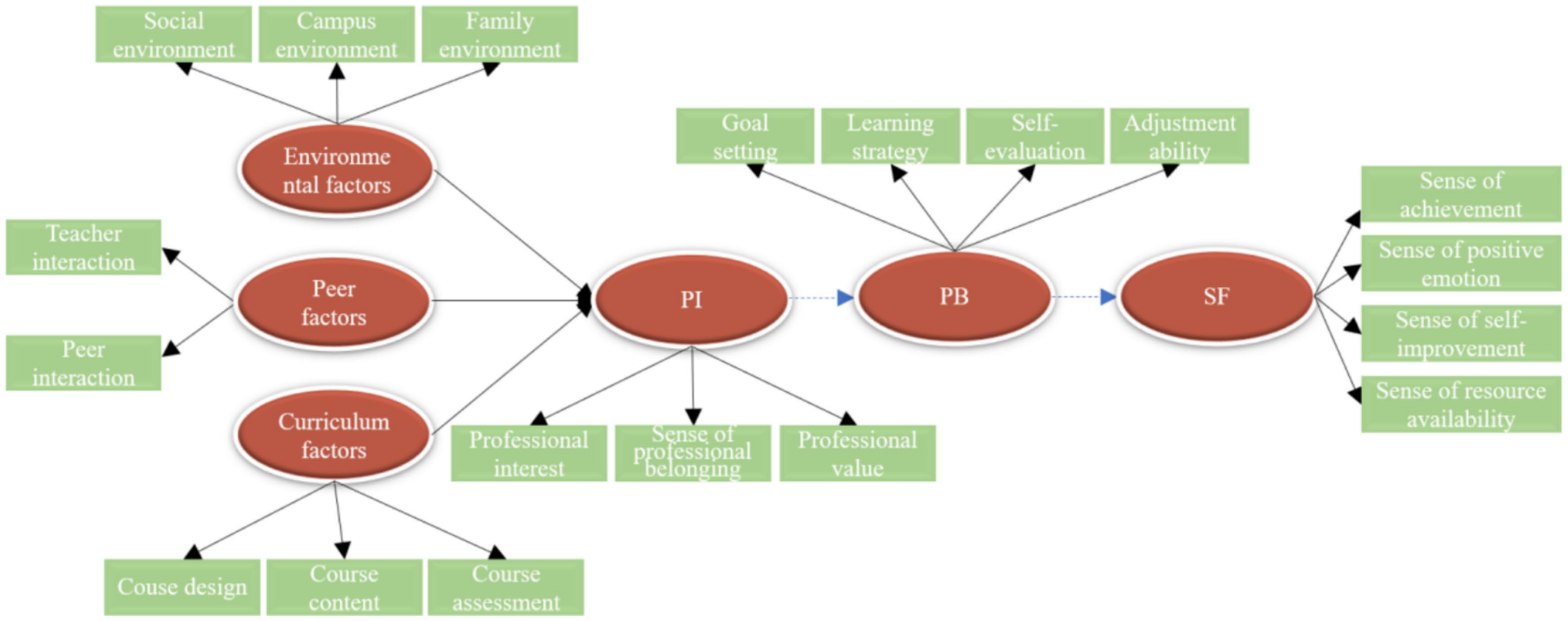
Diagram of theoretical hypothesis model.

Based on the variable specifications outlined above, this study proposes seven research hypotheses. Combined with Diener (1984)’s theoretical framework of SF subjective well-being, it is hypothesized that the deepening of professional cognition, emotion, and value can directly enhance students’ achievement experience (Hypothesis 1). This path is supported by Wood and Locke (1987) about academic self-efficacy theory, indicating that students with high professional identity are more likely to generate positive academic emotions. Based on Cross (2006) research paradigm on the mediating effect of academic achievement, PB is introduced as a mediating variable (Hypotheses 2–4). This design integrates Pintrich and De (1990)’s theory of learning engagement, emphasizing the bridging role of active learning at the behavioral level (such as extracurricular practice and innovative activities) in transforming professional identity into practical acquisition, echoing Chen et al. (2022)’s key finding on the impact of the practice participation dimension on SF. Referencing Henning (2001) research on the interaction between personal traits and professional environment and Yazdannik et al. (2012)’s conclusion on the influence of social status on professional identity, this study constructs the driving paths of environmental factors (Hypothesis 5), peer factors (Hypothesis 6), and curriculum factors (Hypothesis 7) on PI. In particular, Li et al. (2011) find that autonomous professional choice enhances identity provides direct evidence for the hypothesis about the influence of curriculum factors. The theoretical model is illustrated in Figure 2.

*H1*: PI has a direct positive effect on SF.

PI plays a pivotal role in fostering students’ active involvement and participation in their studies (Zhang and Li, 2018) and its formation process is influenced by factors such as the environment, peers, and curriculum (Wang and Geng, 2013). For mining engineering students, the development of PI is particularly significant in influencing SF. When individuals cultivate PI toward their major, they are more likely to develop interest and commitment, which in turn reinforce SF. PI also provides emotional support, fostering a sense of belonging and promoting the establishment of professional values. This support enables individuals to better confront academic difficulties and challenges, mitigating stress and strengthening SF. Furthermore, PI motivates individuals to achieve academic accomplishments, which are inherent manifestations of SF. Individuals with stronger PI tend to perceive academic achievements as reflections of their personal capabilities and worth, thereby enhancing their SF.

*H2*: PI exerts a direct positive influence on PB.

PI stimulates individual action and directly impacts goal-setting processes. When individuals possess PI, they are more likely to set clear, major-related goals and plans, both short-term and long-term. PI also fosters greater motivation to achieve these goals and plans. With clear goals in place, individuals devise learning strategies tailored to their capabilities, including scientific and rational methodologies, facilitating the effective acquisition of professional knowledge and skills. Individuals with high PI tend to engage in positive self-evaluation, objectively assess their goal-setting, academic performance, and progress, and are more willing to accept feedback for improvement. Additionally, PI influences individuals’ adaptability to a certain extent. Those who identify with their major demonstrate enhanced resilience and adaptability when facing academic difficulties or challenges, enabling them to overcome setbacks and obstacles by adjusting their mindset and seeking help.

*H3*: PB exerts a direct and positive influence on SF.

PB is a critical factor influencing the formation of SF. The realization of set goals and plans lays a solid foundation for the development of SF, as goal achievement inherently fosters a sense of accomplishment. Objective and rational learning strategies, coupled with self-assessment, enable individuals to receive favorable learning feedback, creating a virtuous cycle between learning behavior and feedback. This, in turn, helps individuals maintain a positive emotional state. Additionally, the perceived abundance of professional resources influences individuals’ sense of resource availability. The combined effects of goal-setting, learning strategies, self-assessment, and adaptability involved in PB contribute to enhancing individuals’ senses of achievement, positive emotion, self-improvement, and resource availability in their studies.

*H4*: PI has an indirect positive effect on SF by stimulating PB.

While PI and SF are both subjective emotional cognitions, their interaction is often mediated by behavioral factors. Individuals with high PI are more willing to participate in academic activities related to their major. As they develop positive cognitions about these activities, they are likely to experience SF. The corresponding actions taken by individuals, i.e., PB, serve as a mediating factor between PI and SF. PI can stimulate students to engage in PB, further fulfilling their SF.

*H5*: Environmental factors exert a positive influence on PI.

Environmental factors, as the background of individual learning and life, play a critical role in the formation and development of PI. The campus environment, including teaching facilities, faculty strength, and learning atmosphere, provides material and intellectual support for students’ professional studies. Institutions offering ample learning resources, practical opportunities, and a vibrant academic environment foster positive cognitions toward students’ majors. Campus culture and extracurricular activities also influence PI. Participation in professional-related cultural events and club organizations deepens students’ understanding of their majors, fostering a sense of belonging and enhancing PI. Additionally, fluctuations in the external job market and shifts in market demands impact PI. Majors with promising career prospects and strong market demands are more likely to be valued and identified with by students.

*H6*: Peer factors exert a positive influence on PI.

Peer factors, or the mutual influence between classmates, play a significant role in the formation and consolidation of PI. University students frequently interact with peers, and their ideas, attitudes, and behaviors often influence one another reciprocally. When classmates hold positive attitudes toward their majors and demonstrate high enthusiasm and commitment, such a positive peer atmosphere subtly influences students, fostering greater curiosity and interest in their majors. Peer interactions and academic exchanges also provide crucial opportunities for accessing professional information and broadening horizons. These experiences facilitate deeper understanding and identification with their major. Furthermore, mutual support and encouragement among peers assist students in overcoming academic difficulties, enhancing their professional self-confidence and sense of belonging.

*H7*: curriculum factors positively influence PI.

Curriculum factors, a core component of the education system, significantly shape students’ PI. Firstly, the practicality and relevance of course content enable students to perceive the utility of their studies for future careers, enhancing their sense of identity and value toward the profession. Secondly, interactive teaching methods, such as case analysis, project-based learning, and group discussions, stimulate learning interest and initiative, deepening students’ understanding of professional knowledge and fostering a sense of enjoyment and challenge in learning. Finally, the fairness and incentives of the course evaluation system reinforce PI. A scientific and comprehensive evaluation system that accurately reflects academic performance helps students perceive a positive correlation between effort and reward, bolstering their motivation and self-confidence. Recognition and affirmation through evaluations remarkably strengthen students’ sense of belonging and pride.

## 4 Research design

### 4.1 Data sources

**Figure 3:**
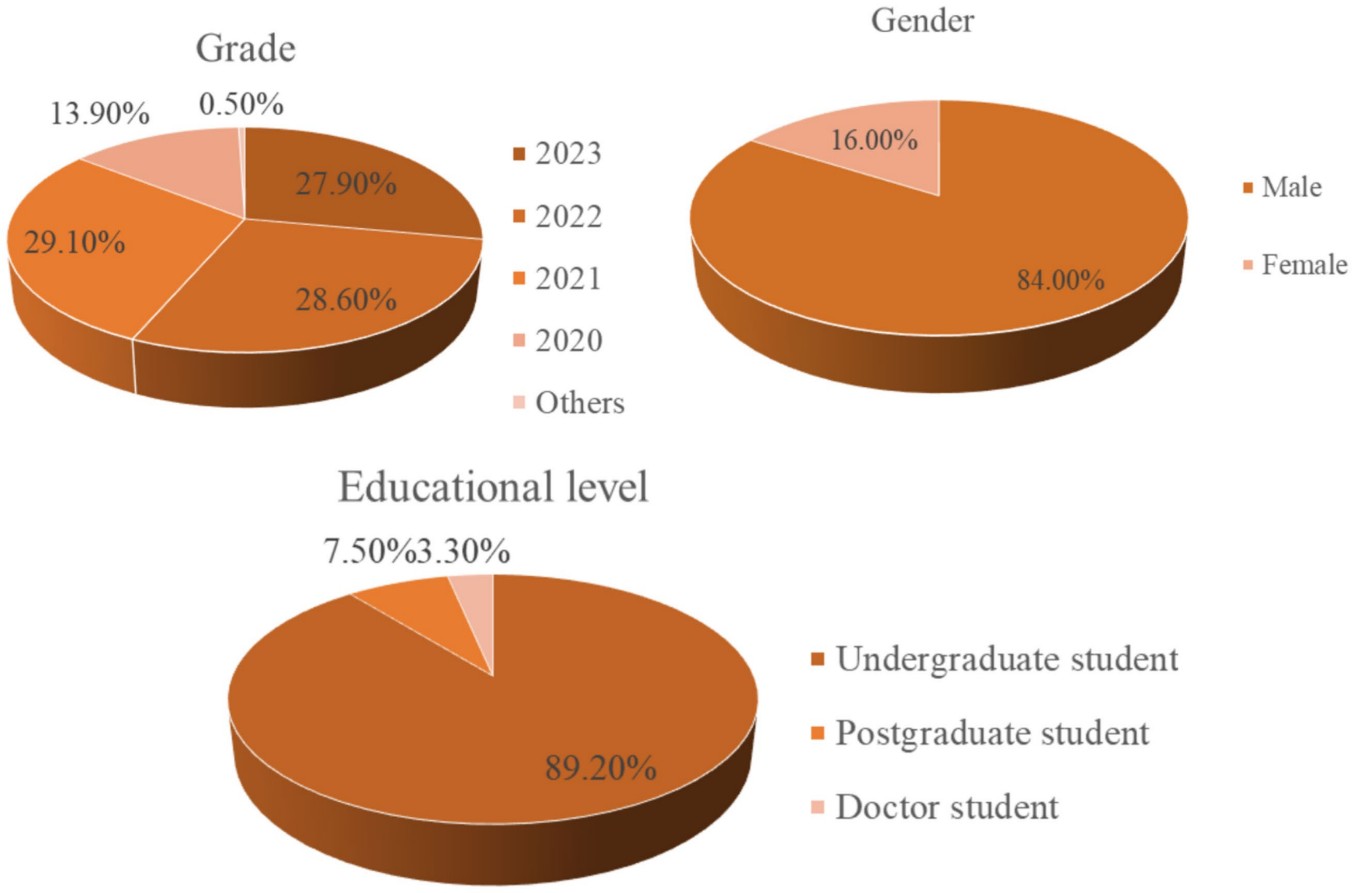
Diagram of sample type pie.

This study employed a survey questionnaire distributed to college students majoring in mining across the country. Participants were categorized based on multiple dimensions, including grade, gender, educational level, professional direction, grade point average (GPA), and family income. A total of 1,007 valid questionnaires were collected, serving as the primary data source for this research. The demographic distribution of the sample is detailed in Table 1, and the sample composition is visually represented in Figure 3.

**Table 1:** Characteristic distribution of data samples.

Dimension	Choice	Frequency	Percentage
Grade	2023	281	27.9%
	2022	288	28.6%
	2021	293	29.1%
	2020	140	13.9%
	Others	5	0.5%
Gender	Male	846	84.0%
	Female	161	16.0%
Educational level	Undergraduate student	898	89.2%
	Postgraduate student	76	7.5%
	Doctor student	33	3.3%
Professional direction	Mining	889	88.3%
	Other engineering majors	112	11.1%
	Non-engineering major	6	0.6%
GPA	4.0 or above	58	5.8%
	3.5–3.99	212	21.1%
	3.0–3.49	347	34.5%
	2.5–2.99	295	29.3%
	2.49 or less	95	9.4%
Students from poor families	Yes	347	34.5%
	No	660	65.5%
Total		1,007	100.0%

### 4.2 Scale design

The research model specifies PI, environmental factors, peer factors, and curriculum factors as independent variables; SF as the dependent variable; and PB as the mediating variable.

The observable variables for PI include professional interest, sense of belonging, and professional value. Environmental factors are operationalized through social environment, campus environment, and family environment. Peer factors are measured by teacher-student interaction and peer interaction, while curriculum factors are assessed through course design, course content, and course assessment.

SF, the dependent variable, is measured by four observable variables: sense of achievement, positive emotion, self-improvement, and resource availability. Sense of achievement reflects individuals’ subjective experience of goal attainment and self-evaluation. Positive emotion captures the emotional states following goal achievement. Self-improvement represents individuals’ subjective assessment of their progress through learning and experience, while resource availability reflects their evaluation of the quality and quantity of accessible learning resources.

PB, the mediating variable, is measured by four observable variables: goal setting, learning strategy, self-assessment, and adjustment capability. Goal setting, guided by goal-setting theory, involves the clarification of learning directions and the establishment of periodic tasks. Learning strategies encompass the methods and techniques employed during the learning process, directly impacting learning outcomes. Self-assessment, as a manifestation of metacognitive ability, involves reflection and evaluation of one’s learning process and results. Adjustment capability refers to individuals’ ability to adapt their emotions, behaviors, and learning strategies in response to challenges and changes, serving as a key indicator of learning adaptability and stability.

The questionnaire utilized a Likert 5-point scale for measurement. The specific variables and corresponding measurement items are detailed in Table 2.

**Table 2:** Variables and corresponding measurement items.

Variable	Observable variable	Item
PI	Professional interest	A11: I have a keen interest in learning professional theoretical knowledge. A12: I am looking forward to participating in some practical activities related to my major.
	Sense of belonging	A21: I am proud of my major. A22: I feel like being part of this professional group.
	Professional value	A31: I recognize my professional role and industry culture. A32: I am willing to recommend my major to others. A33: I am satisfied with my major.
SF	Sense of achievement	B11: I have achieved satisfactory academic performance during the study. B12: I have participated in professional related competitions or projects, and achieved good results. B13: I have won many scholarships through learning and practice. B14: I can find satisfactory jobs related to my major.
	Sense of positive emotion	B21: I will feel satisfied and proud when I complete a difficult academic task B22: I will feel bad when I find that I am inferior to others in terms of professional skills. B23: I will feel that the effort is not in vain when the set academic goals are achieved.
	Sense of self-improvement	B31: I will actively seek help when I encounter difficulties in my study. B32: I can quickly integrate into the team to complete a job together. B33: I am good at using my professional knowledge to overcome difficulty.
	Sense of resource availability	B41: My teachers or supervisors often help students resolve learning difficulty. B42: I can learn much professional knowledge or experience from my teachers. B43: The professional learning atmosphere in my college is quite strong.
Environmental factor	Social environment	C11: The current professional employment environment affects my professional choice. C12: The society has a positive evaluation and support for my major.
	Campus environment	C21: The cultural environment of the campus positively affects my PI. C22: The academic and cultural atmosphere of the campus affects my PI. C23: The level of subject platform and learning resources affect my PI.
	Family environment	C31: I think the economic background of my family will affect my professional choice. C32: I believe the occupation and advice of my family will affect my PI.
Peer factor	Teacher-student interaction	D11: I think teachers should be the collaborators and guides of students. D12: The guidance of professional teachers has enhanced my understanding and recognition of the major. D13: The positive feedback from my teachers makes me more motivated for professional learning.
	Peer interaction	D21: Friends and classmates’ evaluation regarding my major affects my professional choice. D22: The competition and cooperation between classmates can stimulate my interest in professional learning. D23: I like discussing professional knowledge and professional development prospects with my classmates.
Curriculum factor	Course design	E11: Learning theory courses helps me to understand the major. E12: Practice experience will affect my understanding of the major.
	Course content	E22: The professional knowledge and skills provided by the course are quite helpful for me to understand the major. E23: The practicality and applicability of the course can help me understand my major.
	Course assessment	E31: A reasonable course assessment method can enhance my interest in the major.
PB	Goal setting	F11: I have a clear career goal and work hard to achieve it. F12: I have a clear academic goal and work hard to achieve it.
	Learning strategy	F21: I will explore suitable learning strategies to improve my learning efficiency. F22: Learning methods and habits will directly affect my learning results.
	Self-assessment	F31: I can evaluate the learning effect reasonably according to the learning situation and adjust the learning method accordingly. F32: I am able to manage my study time and resources effectively.
	Adjustment capability	F41: I remain optimistic when the learning results and efforts are not proportional. F42: I often reflect on my learning state and make positive adjustments. F43: I maintain a high self-motivation for my academic progress.

### 4.3 Reliability and validity analysis of scales

#### 4.3.1 Reliability analysis

The internal consistency of the scale was assessed using Cronbach’s Alpha coefficient. As shown in Table 3, the Cronbach’s Alpha coefficients for all dimensions and the overall scale ranged from 0.75 to 1, indicating excellent internal consistency and reliability.

**Table 3:** Reliability analysis on the scale.

Dimension scale	Cronbach’s Alpha	Number of terms
PI	0.91	3
SF	0.841	4
Environmental factor	0.77	3
Peer factor	0.805	2
Curriculum factor	0.904	3
PB	0.915	4
Effect of PI on SF	0.962	19

#### 4.3.2 Validity analysis

The validity of the scale was evaluated through multiple indices. As presented in Table 4, the CMIN/DF (Chi-Square to Degrees of Freedom ratio) was 1.414, falling within the acceptable range of 1 to 3. The RESEA (Root Mean Square Error of Approximation) was 0.038, below the threshold of 0.05. Additionally, the IFI (Incremental Fit Index), TLI (Tucker-Lewis Index), and CFI (Comparative Fit Index) all exceeded 0.9, indicating a strong fit of the SEM model to the proposed SF model.

**Table 4:** Model adaptability test.

Index	Reference standard	Test result
CMIN/DF	1–3: excellent; 3–5: good	1.414
RMSEA	<0.05: excellent; <0.08: good	0.038
IFI	>0.9: excellent; >0.8: good	0.986
TLI	>0.9: excellent; >0.8: good	0.982
CFI	>0.9: excellent; >0.8: good	0.985

Under the premise of good model fit, the average variance extracted (AVE) and composite reliability (CR) were further examined. The factor loadings of the measurement items were computed through the established SEM model. According to the mathematical calculation methods of engineering statistics, the AVE and CR values were derived using Equations (1) and (2):


(1) $$\mathrm{AVE}=\frac{\sum \lambda^{2}}{\sum \lambda^{2}+\sum \theta }$$

(2) $$\mathrm{CR}=\frac{{\left(\sum \lambda \right)}^{2}}{{\left(\sum \lambda \right)}^{2}+\sum \theta }$$

(3) $$\begin{array}{c} \theta =1-\lambda^{2} \end{array}$$

In Equations (1) – (3), λ represents the factor loading, which is the standardized regression coefficient from the latent variable to the measured variable. A larger factor loading indicates that the latent variable has a stronger ability to explain the measured variable, meaning better reliability of the indicator. λ^2^ is called the reliability coefficient, which represents the square of the factor loading and is equivalent to the R-squared value of the univariate linear regression equation established between the measured variable (dependent variable) and the latent variable (independent variable). A higher reliability coefficient signifies a stronger explanatory power of the latent variable for the measured variable and better reliability of the indicator. In Equation (3), *θ* denotes the measurement error. A larger reliability coefficient implies a smaller measurement error, indicating a stronger explanatory power of the latent variable for the measured variable and better indicator reliability.

As shown in Table 5, the AVE values for all dimensions exceeded 0.5, and the CR values surpassed 0.7, demonstrating strong convergent validity and composite reliability across all dimensions.

**Table 5:** Convergence validity and composite reliability in various dimensions of the scale.

Path relationship	Estimate	AVE	CR
HJ1 ← Environmental factor	0.892	0.605	0.818
HJ2 ← Environmental factor	0.785		
HJ3 ← Environmental factor	0.634		
PB1 ← Peer factor	0.752	0.621	0.766
PB2 ← Peer factor	0.822		
KC1 ← Curriculum factor	0.736	0.576	0.802
KC2 ← Curriculum factor	0.844		
KC3 ← Curriculum factor	0.689		
ZR1 ← PI	0.583	0.594	0.810
ZR2 ← PI	0.849		
ZR3 ← PI	0.849		
GT1 ← PB	0.659	0.538	0.822
GT2 ← PB	0.695		
GT3 ← PB	0.796		
GT4 ← PB	0.775		
XY1 ← SF	0.825	0.502	0.800
XY2 ← SF	0.632		
XY3 ← SF	0.644		
XY4 ← SF	0.717		

## 5 Case study

### 5.1 Descriptive statistics and normality tests

This study employs a 5-point Likert scale for all measurements. The descriptive statistics for each dimension and the normality test results for the measurement items are presented in Table 6. According to the descriptive statistical analysis (Table 6), the mean scores for all variables range between 3 and 4, indicating a moderate to high level of agreement across all measured constructs.

**Table 6:** Descriptive statistics in various dimensions and normality test results of measurement items.

Dimension		Average value	Standard deviation	Skewness	Kurtosis	Population mean	Population standard deviation
PI	ZR1	3.738	0.793	−0.970	1.980	3.735	0.769
	ZR2	3.788	0.845	−0.931	1.582		
	ZR3	3.680	0.866	−0.686	0.783		
SF	XY1	3.057	0.895	0.241	−0.282	3.670	0.620
	XY2	3.888	0.676	−1.152	3.580		
	XY3	3.826	0.707	−0.787	2.221		
	XY4	3.907	0.717	−0.835	2.187		
Environmental factor	HJ1	3.772	0.750	−0.727	1.790	3.826	0.632
	HJ2	3.918	0.733	−0.934	2.279		
	HJ3	3.790	0.805	−0.837	1.348		
Peer factor	PB1	4.100	0.653	−0.978	3.256	3.969	0.618
	PB2	3.838	0.698	−0.708	1.746		
Curriculum factor	KC1	4.049	0.645	−0.925	2.992	4.029	0.635
	KC2	4.025	0.678	−1.018	3.179		
	KC3	4.013	0.752	−1.160	3.015		
PB	GT1	3.811	0.779	−0.729	1.356	3.877	0.654
	GT2	4.039	0.656	−0.967	3.205		
	GT3	3.859	0.741	−0.705	1.406		
	GT4	3.797	0.750	−0.738	1.671		

Normality tests for the measurement items were conducted using skewness and kurtosis coefficients. The results show that the absolute values of skewness and kurtosis comply with the criteria proposed by Kline (2016), where skewness ≤ 3 and kurtosis ≤ 8. This confirms that the data approximate a normal distribution, satisfying the assumptions for subsequent statistical analyses.

### 5.2 Pearson correlation analysis

Pearson correlation analysis was conducted to examine the relationships among the variables. As shown in Table 7, all variables exhibit significant correlations. The correlation coefficients between all variables are positive, indicating a strong and consistent relationship among the constructs under investigation. These findings provide preliminary support for the hypothesized relationships in the proposed model.

**Table 7:** Pearson correlation analysis results among various variables.

Dimension	PI	SF	Environmental factor	Peer factor	Curriculum factor	PB
PI	1					
SF	0.764^**^	1				
Environmental factor	0.642^**^	0.753^**^	1			
Peer factor	0.656^**^	0.768^**^	0.788^**^	1		
Curriculum factor	0.660^**^	0.730^**^	0.698^**^	0.822^**^	1	
PB	0.663^**^	0.772^**^	0.708^**^	0.764^**^	0.784^**^	1

^**^The correlation is significant at the level of 0.01 (two-tailed).

### 5.3 SEM analysis

#### 5.3.1 Path relationship hypothesis testing

The SEM analysis was conducted to test the hypothesized relationships among the variables. The results, as presented in Table 8, reveal the following:

- Hypothesis 1: PI has a significant positive effect on SF (*β* = 0.562, *p* < 0.001). This hypothesis is supported.
- Hypothesis 2: PI significantly influences PB (*β* = 0.790, *p* < 0.001). This hypothesis is supported.
- Hypothesis 3: PB significantly impacts SF (*β* = 0.425, *p* < 0.001). This hypothesis is supported.
- Hypotheses 5, 6, and 7: Environmental factors, peer factors, and curriculum factors do not demonstrate a significant direct effect on PI (*p* > 0.05). These hypotheses are not supported.

**Table 8:** Test results of SEM path relationship among influencing factors of SF.

Path relationship	Standardized regression coefficient	SE	z (CR value)	*p*
PI → SF	0.562	0.037	13.341	0
PI → PB	0.790	0.035	23.521	0
Environmental factor → PI	−0.876	3.86	−0.243	0.808
Peer factor → PI	0.934	4.053	0.432	0.665
Curriculum factor → PI	−0.989	4.127	−0.379	0.705
PB → SF	0.425	0.031	11.22	0

**Figure 4:**
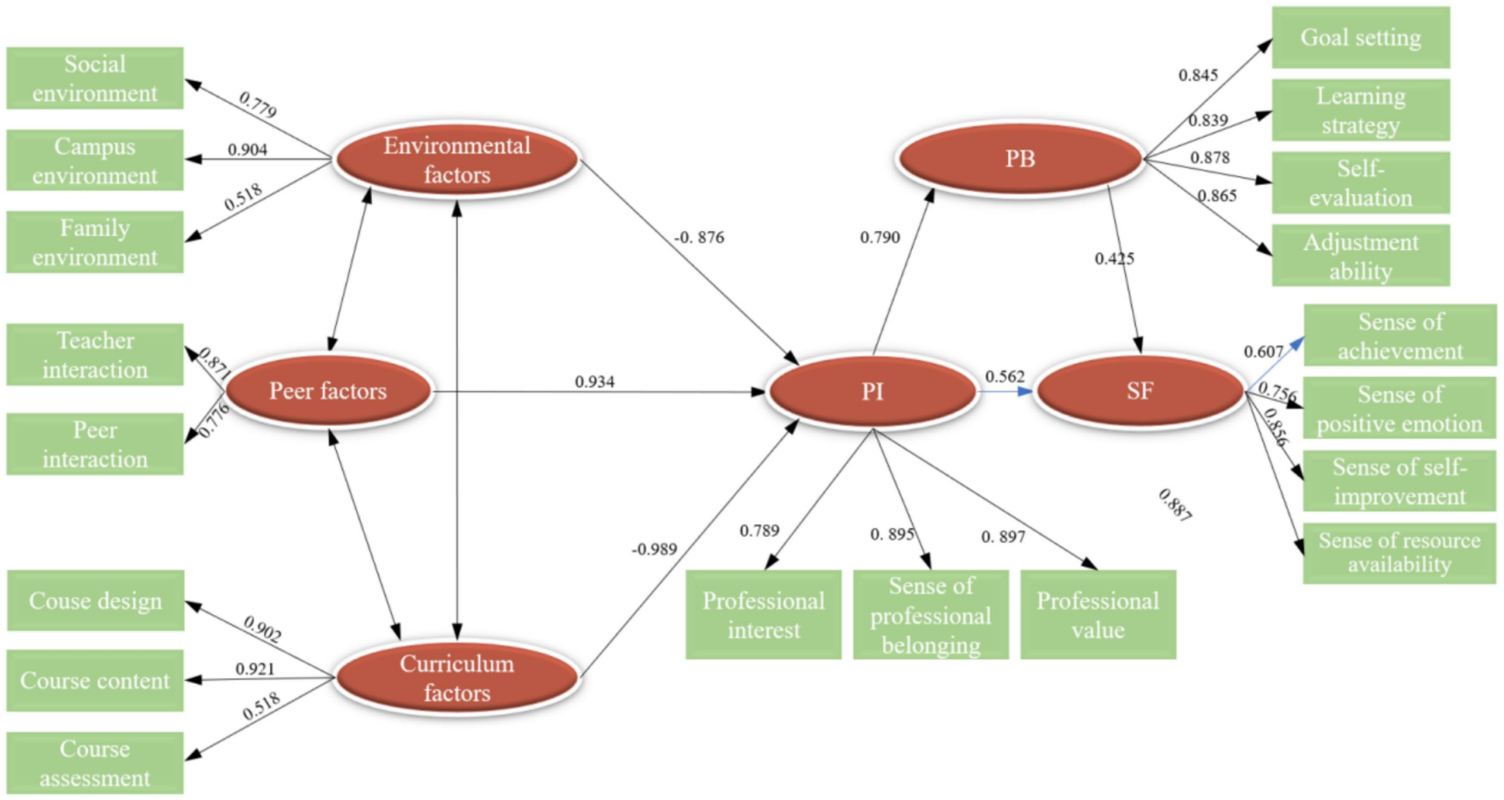
Diagram of the SEM model illustrating the relationship between PI and SF.

The SEM model illustrating the relationship between PI and SF is depicted in Figure 4.

#### 5.3.2 Analysis of the mediating role of PB

The mediating effect of PB between PI and SF was analyzed. The results, as presented in Table 9, indicate that PB serves as a partial mediator, with the mediating effect falling within a 95% confidence interval (c’ ≠ 0). This finding is statistically significant, supporting Hypothesis 4.

**Table 9:** Results of the mediating role of PB.

Item	Symbol	Effect	Effect value	95% CI		SE	z/t	*p*	Conclusion
				Lower limit	Upper limit				
ZR= > GT= > XY	a*b	Indirect effect	0.253	0.262	0.367	0.026	9.557	0	Partial mediation
ZR= > GT	a	X= > M	0.564	0.525	0.604	0.02	28.063	0	
GT= > XY	b	M= > Y	0.449	0.406	0.491	0.022	20.812	0	
ZR= > XY	c’	Direct effect	0.363	0.327	0.399	0.018	19.797	0	
ZR= > XY	c	Gross effect	0.616	0.584	0.648	0.016	37.516	0	

^*^ Indicates significance, a*b represents the mediation effect, showing that the results here support that GT plays a mediating role in the path from ZR to XY.

## 6 Conclusions and limitations

### 6.1 Conclusion

This study constructed a SEM framework to investigate the complex interplay among PI, PB, SF, and external factors (environmental, peer, and curriculum factors) among mining engineering students. The findings reveal that PI, as the core variable, plays a pivotal role in enhancing SF. Specifically:

(1) Direct effect of PI on SF: PI significantly influences SF (*β* = 0.562, *p* < 0.001), confirming its role as a crucial intrinsic driver of SF. PI also serves as a key motivator for positive PB, which in turn reinforces SF.
(2) Mediating role of PB: PB acts as a partial mediator between PI and SF, highlighting the importance of guiding and regulating students’ behaviors to enhance their SF.
(3) Indirect influence of external factors: While environmental, peer, and curriculum factors do not exhibit a direct effect on PI, they may indirectly influence PI through alternative pathways or in conjunction with other variables.

These findings underscore the centrality of PI and PB in fostering SF, while also highlighting the complex and nuanced role of external factors.

### 6.2 Implications and recommendations

Based on the findings, the following recommendations are proposed to enhance students’ PI, PB, and SF:

Strengthen PI education: Higher education institutions should develop comprehensive, multifaceted systems to motivate and support students. This can be achieved through innovative course teaching, practical experiences, and career guidance to help students deeply understand professional values, career prospects, and the alignment between their personal interests and their chosen major.

Foster positive PB: Universities should address students’ needs and encourage them to explore professional goals, curriculum design, and employment prospects while engaging in practical activities. Empirical evidence suggests that experiential learning is more effective than ideological education in shaping positive behaviors. Successful practical experiences can enhance SF by fostering a sense of accomplishment and growth.

Optimize the external cultural environment: Although environmental factors do not directly influence PI, their subtle impact on students’ overall learning experiences cannot be overlooked. Universities should continuously improve campus culture, teaching facilities, and other infrastructural aspects to create a more conducive learning environment.

Leverage peer influence: While peer factors do not directly affect PI, peer interactions and role modeling can significantly contribute to students’ development. Universities can organize experience-sharing sessions and recognize outstanding students to amplify the positive impact of peer dynamics.

Advance curriculum reforms: Although curriculum factors do not directly influence PI, they play a critical role in regulating students’ learning interest and motivation. Universities should prioritize curriculum innovation to ensure courses are advanced, practical, and engaging. Additionally, integrating engineering ethics education and strengthening ethical norms, personnel training systems, and moral supervision mechanisms can further enhance students’ professional development.

Establish a comprehensive evaluation system: Universities should move beyond grade-based assessments and adopt a multidimensional evaluation system that incorporates academic performance, practical skills, emotional attitudes, and other dimensions to comprehensively assess students’ growth and development.

Enhance psychological intervention and counseling: Given the potential psychological challenges arising from PI formation or academic pressure, universities should establish robust psychological consultation and counseling systems to provide timely support and interventions.

### 6.3 Limitations and future research directions

This study has several limitations. First, the sample size and representativeness may influence the generalizability of the findings. Future research could explore larger, more diverse samples to validate the results. Second, the study primarily focuses on direct and mediating effects, leaving the potential indirect effects of environmental, peer, and curriculum factors unexplored. Future studies could investigate how these external factors interact with internal psychological variables, such as learning motivation and self-efficacy, to collectively influence PI and academic performance.

Moreover, the interplay between PI, PB, and SF is complex and multifaceted. Future research could delve deeper into the underlying mechanisms and identify additional mediating or moderating variables using advanced measurement tools and methodologies. Finally, with the rise of educational informatization, digital and intelligent educational methods hold great potential for enhancing students’ PI and SF. Future studies could explore how these emerging technologies transform learning styles and experiences, ultimately fostering students’ capabilities for sustainable development.

## Data Availability

The original contributions presented in the study are included in the article/supplementary material, further inquiries can be directed to the corresponding author.
